# Nanoplate based digital PCR assay for effective quantification of plasma HPV circulating tumor DNA

**DOI:** 10.1038/s41698-026-01424-y

**Published:** 2026-04-21

**Authors:** Preetiparna Parida, Gayathri Baburaj, Ajay A. Shettigar, Krishna Sharan, Mehta Vedant Kamal, Pranav P V, Jayashree N. P, Naveena A. N. Kumar, Ganesh Mohan, Shamee Shastry, Mahadev Rao, Shirley Lewis, Rama Rao Damerla

**Affiliations:** 1 Department of Medical Genetics, Kasturba Medical College, Manipal Academy of Higher Education, Manipal, India; 2 Department of Pharmacy Practice, Center for Translational Research, Manipal College of Pharmaceutical Sciences, Manipal Academy of Higher Education, Manipal, India; 3 Department of Radiotherapy and Oncology, KS Hegde Medical Academy (KSHEMA), Nitte (Deemed to be University), Deralakatte, Mangaluru, India; 4 Department of Surgical Oncology, Kasturba Medical College, Manipal Academy of Higher Education, Manipal, India; 5 Department of Radiation Oncology, Kasturba Medical College, Manipal Academy of Higher Education, Manipal, India; 6 Department of Immunohematology and Blood Transfusion, Kasturba Medical College, Manipal Academy of Higher Education, Manipal, India

**Keywords:** Biological techniques, Biomarkers, Biotechnology, Cancer, Oncology

## Abstract

High risk Human papillomavirus (hrHPV) is a leading cause of cervical cancer. Numerous studies have demonstrated the utility of plasma HPV circulating tumor DNA (ctDNA) for disease monitoring in cervical cancer, nevertheless with diverse accuracies. We investigated whether higher sample and reaction volumes using nanoplate based digital PCR (dPCR) system offered enhanced sensitivity and reproducibility, crucial for detecting low-copies of HPV ctDNA. A multiplex dPCR assay was optimized for detecting hrHPV16, 18, and 31 plasma ctDNA from pre-treatment and follow-up plasma samples of 87 cervical cancer patients using Qiagen QIAcuity One dPCR. The assay demonstrated 98% sensitivity and 100% specificity for detecting HPV ctDNA from pre-treatment plasma cell-free DNA (cfDNA). Persistence of HPV ctDNA during follow up associated with disease relapse, while clearance of HPV ctDNA was observed in patients without recurrence. Higher sample and dPCR reaction volumes increased the sensitivity of the test, while HPV ctDNA monitoring effectively reflected disease burden. **Trial registration:** Clinical Trials Registry of India (CTRI/2020/01/022862); registered on 20 January 2020.

## Introduction

Cervical cancer remains a substantial global health challenge, ranking as the fourth most common cancer among women worldwide^1^. A vast majority of cervical cancer cases are driven by persistent infections with high-risk human papillomavirus (hr-HPV) types, particularly HPV16 and HPV18^2^. Despite advancements in screening methods like Pap smears and HPV DNA testing, challenges persist in early detection and monitoring treatment response, particularly in resource-limited settings^3,4^.

Circulating cell-free DNA (cfDNA) has emerged as a promising non-invasive biomarker that helps in detection and tracking disease progression in cancer. Circulating cfDNA, including HPV-derived cfDNA, is released into the bloodstream from tumor cells undergoing apoptosis or necrosis. CfDNA in healthy individuals is derived from hematopoietic cells, but in the case of patients with cancer, it is usually derived from tumor cells^5^. As reported by previous studies, HPV circulating tumor DNA (ctDNA) has found utility in diagnosis, prediction of relapse, and treatment monitoring of cervical cancer with remarkable sensitivity and specificity^6–8^. However, detecting low-abundance HPV DNA in blood is inherently difficult due to the extremely low concentration of viral genetic material present. While next generation sequencing (NGS) is the most preferable method for comprehensive cfDNA characterization, it presents drawbacks such as high cost, complex analysis, and a lengthy turnaround time^9^. The most commonly used method for detection of cfDNA is currently droplet digital PCR (ddPCR), which relies on generation of thousands of droplets by splitting the PCR in an oil emulsion, enabling absolute quantification of target sequences^9^. Several studies have reported the utility of Bio-Rad’s QX droplet digital PCR systems to analyse cfDNA in cervical cancer and have demonstrated the potential of cfDNA monitoring in prediction of recurrence, surveillance of treatment response, and assessment of clinical outcomes^6–8,10–12^. However, these systems have inherent drawbacks, some of which include the need for multiple equipment to generate, amplify, and read droplets, droplet variability in size and robustness, and possible contamination due to multiple transfer steps involved. The new nanoplate-based digital PCR systems present solutions to address these limitations. These systems utilize microfluidic nanoplate technology to partition reactions into approximately 26,000 uniform nano-wells within a fully enclosed, automated workflow. This design minimizes sample handling and contamination risks, enhances reproducibility, and supports higher throughput. Importantly, the ability to accommodate larger reaction volumes increases sensitivity, which is critical for the detection of low-concentration targets in the samples^9^.

The aim of this study was to validate the novel nanoplate-based dPCR system for the detection and treatment monitoring of cervical cancer patients using HPV ctDNA as a marker. By analysing HPV ctDNA levels at various stages of treatment, we aim to assess their correlation with treatment response, disease remission, and relapse. This approach has the potential to provide valuable insights into patient prognosis and improve personalized treatment strategies by enabling earlier intervention in cases of disease recurrence.

## Results

### Multiplex PCR optimisation for HPV subtypes 16, 18 and 31

We first standardized primers to amplify E6 sequences from all three HPV subtypes 16, 18, and 33 at a common annealing temperature of 55 °C (supplementary Fig. 1). Specificity analysis of the multiplex HPV qPCR assay demonstrated no cross-reactivity, as each primer-probe set was able to detect the target HPV type without amplifying spiked-in non-target backgrounds. The HPV 16 assay yielded positive results only in the presence of HPV 16, while remaining negative for HPV 18 and HPV 31 backgrounds. Similarly, the HPV 18 assay was specific to HPV 18, and the HPV 31 assay detected only HPV 31, confirming the precision of primers and probes designed for the assay (Supplementary Fig. 2). To further optimise the multiplex assay, different primer: probe concentrations were tested on dPCR. The data suggested an optimal primer to probe ratio of 1.5 μM:1.5 μM, which yielded optimal separation between positive and negative droplet populations on dPCR (supplementary Fig. 3). Cross-reactivity of the multiplex dPCR assay was evaluated using omission matrix mixes in which one HPV genotype was intentionally excluded while the remaining two were included. Across all omission conditions, no false-positive partitions were detected for any primer–assay combination. The HPV16 assay generated positive partitions only in reactions containing SiHa DNA and remained negative in mixes lacking HPV16. Similarly, the HPV18 assay detected signal exclusively in reactions containing HeLa DNA, and the HPV31 assay was positive only in the presence of the HPV31-positive template. All assays remained negative in the corresponding omission mixes and in no-template controls (supplementary Fig. 4). These data demonstrated that the multiplex dPCR design exhibited high analytical specificity, with no evidence of cross-amplification or non-target detection under any tested condition.

Limit of Blank (LoB) was estimated by analysing 20 no template controls measuring fluorescence for all 3 channels corresponding to HPV 16, 18, and 31 using the multiplex dPCR assay. The HPV16 channel showed one positive partition in 7 of the 20 blank reactions and two positive partitions in 1 of the 20 blank reactions. For HPV18, 3 blank reactions each contained a single positive partition, while for HPV31, 2 blank reactions showed one positive partition each (Supplementary Fig. 5). LoB was calculated as 1.36, 0.74, and 0.59 for HPV 16, 18, and 31, respectively (Supplementary file 1). Hence, any ddPCR result with fewer than two positive droplets was considered “negative” for our multiplex dPCR. To evaluate Limit of Detection (LoD), HPV 16, 18, and 31 recombinant plasmids were 10-fold serially diluted, from 10^3^ to 1 copy/μL, as templates and were amplified using E6 primers and probes on dPCR. (supplementary Fig. 6). At the lowest non-zero input (1 copy), the assay detected two positive partitions for HPV16 and three positive partitions for both HPV18 and HPV31. These low-copy detection patterns contributed directly to the LoD calculation in accordance with CLSI EP17 guidelines.

### HPV-ctDNA detection in clinical samples

A total of 87 patient samples were recruited in the study, and their demographics along with clinical profiles are summarised in Table 1. The mean age at diagnosis was 56 years (SD 10.2 years), and median age was 55 years (IQR = 15.5). Most patients presented with stage II (*n* = 41) or stage III (*n* = 33) disease. The most common histological tumor type was Squamous cell carcinoma (SCC) (*n* = 84), followed by adenocarcinoma (*n* = 3). We first determined HPV status of our cohort of 87 patients by MY09/MY11 and nested GP5+/GP6+PCR followed by Sanger sequencing to identify HPV subtypes from tissue biopsies. Among them 75 were positive for HPV16, 6 for HPV18, 2 were positive for HPV 31, while 4 patients were HPV negative (Table 1) (Supplementary Fig. 7). Plasma cfDNA was amplified using HPV ctDNA multiplex dPCR assay and the Sensitivity, specificity, positive predictive value (PPV), and negative predictive value (NPV) were calculated using the tissue HPV result as the reference standard.

**Table 1:** Demographics and clinical characteristics of the patients enrolled in the study

Characteristics	All Patients	HPV ctDNA detectable at diagnosis
Total	87	82
Age (in years)	Mean = 56 years (SD = 10.2 years) Median = 55 years (IQR = 15.5)	
Stage		
I	4	4
II	41	37
III	33	33
IV	9	8
Histological type		
Squamous cell carcinoma	84	79
Adenocarcinoma	3	3
HPV types		
16	75	74
18	6	6
31	2	2
Negative	4	0

Diagnostic performance of cfDNA-based HPV detection was evaluated using the following equations:

1 $${\mathrm{Sensitivity}}=\frac{TP}{TP+FN}=\frac{82}{82+1}=98.8 \%$$

2 $${\mathrm{Specificity}}=\frac{TN}{TN+FP}=\frac{4}{4+0}=100 \%$$

3 $${\mathrm{Positive}}\,{\mathrm{Predictive}}\,{\mathrm{Value}}\,({\mathrm{PPV}})=\frac{TP}{TP+FP}=\frac{82}{82+0}=100 \%$$

4 $${\mathrm{Negative}}\,{\mathrm{Predictive}}\,{\mathrm{Value}}\,({\mathrm{NPV}})=\frac{TN}{TN+FN}=\frac{4}{4+1}=80.0 \%$$

where TP denotes true positives, TN true negatives, FP false positives, and FN false negatives.

**Fig. 1:**
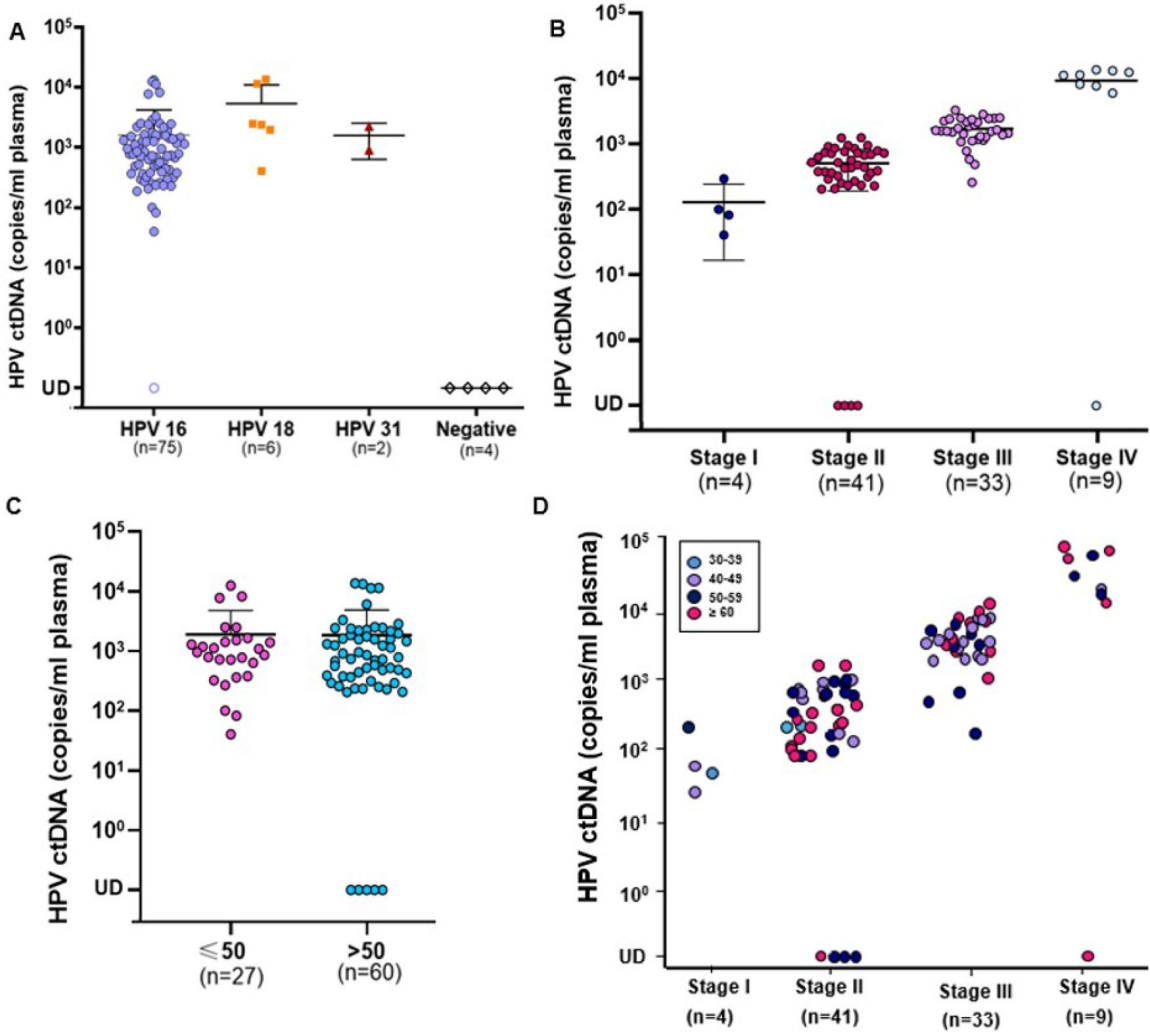
HPV ctDNA detection by dPCR before treatment and correlation with clinical parameters. **A** Sensitivity and specificity of human papillomavirus (HPV) types 16, 18, and 31 digital polymerase chain reaction (dPCR) multiplex assay. Blue dots represent HPV 16 positive on dPCR, orange squares represent HPV 18, and red triangles represent HPV 31 positive samples. The black diamond represents the sample which were negative for HPV on tumor testing. A white dot with a blue outline represents a sample that was positive for HPV 16 by tumor testing but was undetectable in cfDNA samples. (*p* = 0.0006, by Kruskal–Wallis test). **B** HPV ctDNA levels according to cervical cancer stages. (*p* = <0.0001, by Kruskal–Wallis test). **C** HPV ctDNA levels according to age of patients during diagnosis. (*p* = 0.9283 by unpaired *t*-test). **D** HPV ctDNA levels correlated to age and stage. UD Undetermined.

We detected 82 out of 83 samples positive for HPV ctDNA, implying a sensitivity of 98% (Fig. 1), and all the 4 HPV negative samples were negative on HPV ctDNA multiplex dPCR test. All 20 healthy donor samples tested as part negative control experiment were negative, demonstrating 100% specificity (Supplementary Fig. 8). The Positive Predictive Value (PPV) was 100%, and the Negative Predictive Value (NPV) 96%. The median level of HPV ctDNA across all samples was 850 copies/mL plasma (range 0.0–13584), with an interquartile range (IQR) in positive patients of 376.5–1692 copies/mL. We further explored if HPV ctDNA levels from the patients with cervical carcinomas (*n* = 87) correlated with clinical and biological parameters. A significant relationship was also found between disease stage and HPV ctDNA copies (*p* < 0.0001) (Fig. 1B), suggesting that tumor burden may influence the level of detectable HPV ctDNA copies in liquid biopsy. No significant correlation was observed between HPV ctDNA copies and patient age (*p* = 0.9283) (Fig. 1C, D). We also analysed the correlation between HPV ctDNA copy number and gross tumor volume and found no significant correlation with pre-treatment HPV ctDNA levels (Supplementary Fig. 9).

### HPV ctDNA as a marker for therapy response and detection of relapse

**Fig. 2:**
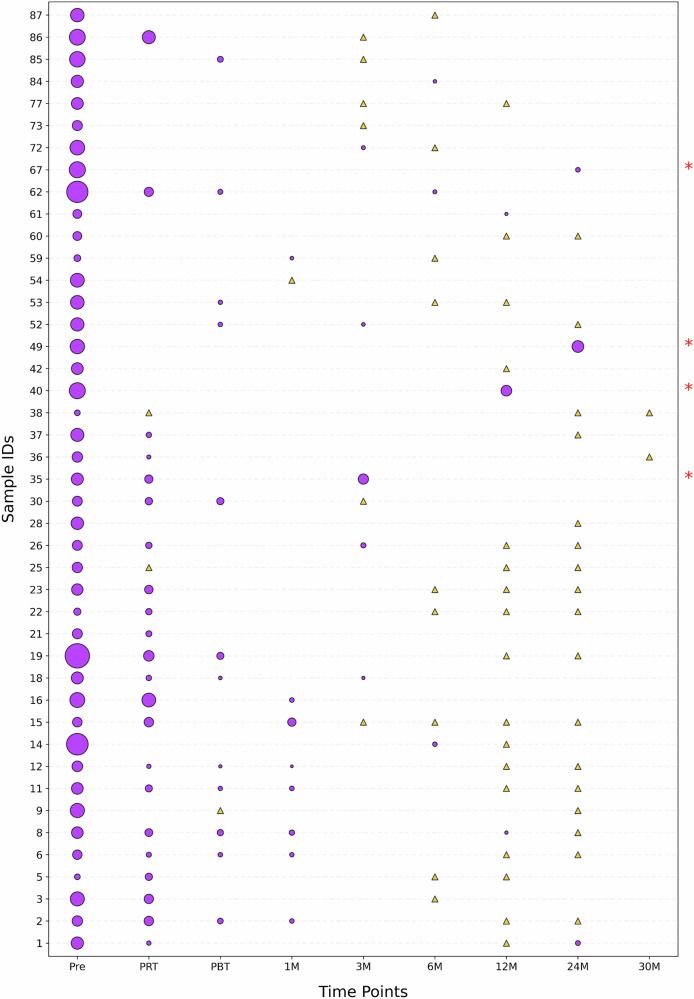
HPV ctDNA dynamics during treatment and follow-up in the prospective cohort. HPV positive samples are represented by circle. The diameter of the circle represents HPV copy number per ml of plasma. Samples with undetectable HPV copies are represented by triangles. Pre-Treatment (PreTX), Post Radiation Therapy (PRT), Post Brachytherapy (PBT), 1 Month (1 M), 3 Months (3 M), 6 Months (6 M), 12 Months (12 M), 24 Months (24 M), 30 Months (30 M). The asterisk (*) denotes patient who experienced relapse during the study period.

**Fig. 3:**
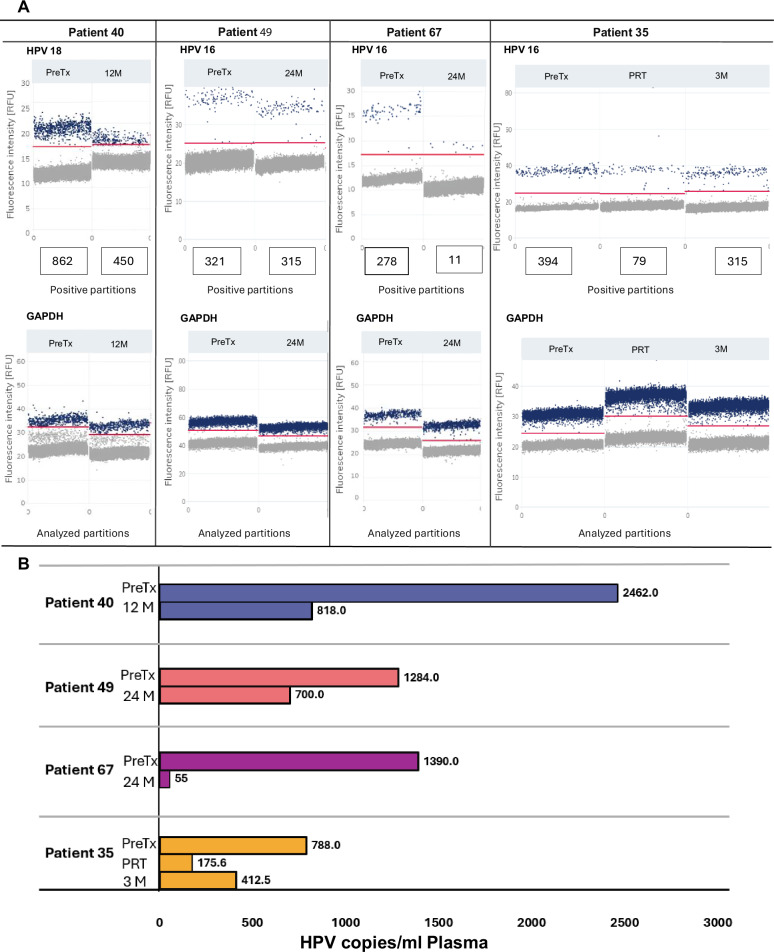
Digital PCR analysis and HPV ctDNA dynamics in patients with relapse. **A** Digital PCR image of patient 40 (HPV 18 positive), showing relapse at 12 M (12 Months) follow up, patient 49 (HPV 16 positive), showing relapse after 24 M (24 Months), patient 67 (HPV 16 positive), showing relapse at 24 M (24 Months) follow up, patient 35 (HPV 16 positive), with follow up at pre-treatment (PreTX), post-radiation therapy (PRT) and 3 months (3 M) showing relapse. **B** Graphs representing the change in levels of HPV ctDNA for 4 patients with relapse.

We obtained longitudinal follow-up samples from 44 patients at various stages of treatment followed by post treatment plasma samples up to 24 months from the time of completion of brachytherapy, which represented our prospective cohort. Most patients exhibited a substantial reduction in HPV ctDNA copies post brachytherapy represented (Fig. 2) treatment or by the 3 month follow up, with levels continuing to decrease by subsequent follow-up visits. (Fig. 2). However, four patients in our cohort presented with relapse, and follow up cfDNA samples from all of them showed detectable levels of HPV ctDNA. We could not collect a complete longitudinal sample profiles for plasma samples from 3 of these relapsed patients. These 3 patients had only two plasma samples available for analysis: an initial pre-treatment baseline sample and a single follow-up sample collected at either the one-year or two-year surveillance timepoint. Patient 40, diagnosed with stage IIIC1 cervical cancer, showed measurable HPV 18 ctDNA levels in the pre-treatment sample (Fig. 3A, B). At the 12-month follow-up post-treatment, the HPV ctDNA remained detectable, although at a lower copy number compared to baseline. The detection of HPV copies at 12 months post therapy also coincided with the patient’s clinical presentation of disease relapse, which was confirmed through clinical examination at the same timepoint. Patient 49, diagnosed with stage IIIC1 disease, also showed detectable amount of HPV 16 copies at the 24 month follow up, post therapy (Fig. 3A, B). The third patient (Patient 67) with stage IIIA disease, also demonstrated an identical pattern, with HPV 16 ctDNA levels detectable in the follow-up timepoint (Fig. 3A, B). The detection of HPV 24 months post-therapy, again coincided precisely with the clinical documentation of disease relapse. A better longitudinal HPV ctDNA profile was obtained for patient 35 with relapse (stage IIB at diagnosis), for whom three plasma samples were collected: pre-treatment, post-radiation therapy (PRT), and at a 3-month follow-up visit. Following radiation, HPV ctDNA levels showed an initial decline. At the 3-month follow-up, however, an increase in HPV ctDNA levels was detected (Fig. 3A, B).

## Discussion

Liquid biopsy technologies are emerging as innovative, minimally invasive approaches in cancer detection, treatment monitoring, and detection of minimal residual disease compared to traditional tissue biopsy^13^. Since almost all cervical cancers are HPV associated, HPV ctDNA emerges as a potential biomarker in cervical cancer^14^. Early studies had used qPCR-based method for detection of HPV cfDNA in cervical cancer, but the sensitivity reported by these studies was very low^15–17^. Recently, with the introduction of ddPCR, studies reported an improved sensitivity and specificity in detection of HPV cfDNA and delineating its role in monitoring treatment response and relapse prediction for patients. Though the specificity of these studies were 100%, the sensitivity ranged between 62-87%^8,10,11,18^.

In this study, we developed a liquid biopsy test using a nanoplate-based dPCR method to enhance the sensitivity of detection of HPV cfDNA in cervical cancer patients. We utilised the Qiagen QIAcuity One system dPCR platform that offers several advantages over droplet-based systems. A key strength this dPCR test lies in its fully automated workflow, which minimizes handling errors, improves consistency, and significantly accelerates turnaround time^19^. The analytical performance of our multiplex dPCR assay was supported by a clearly defined limit of blank (LoB), a low limit of detection (LoD), and the absence of false-positive partitions across all normal control samples, demonstrating 100% assay specificity under our conditions.

The assay demonstrated a PPV of 100%, indicating that all samples classified as HPV-positive by the multiplex dPCR assay were truly positive. This underscores the high analytical specificity of the assay and confirms that a positive result can be interpreted with complete confidence. In contrast, the NPV of 96% reflects that almost all HPV-negative calls were accurate, although a small probability of false-negative detection remains. This is expected in cfDNA-based assays, where extremely low viral copies or sampling stochasticity can occasionally lead to missed detection. Together, these values highlight that while the assay is highly reliable for confirming HPV positivity, caution may be warranted when interpreting negative results in clinically suspected cases, and repeat testing or clinical verification may be warranted in inconclusive situations.

Another major advantage of dPCR is the capacity to accommodate larger sample volume, which is crucial for increasing the sensitivity of cfDNA-based liquid biopsy tests due to low yields of cfDNA extracted from any biofluids^9^. The sample volume in the case of ddPCR is about 7 µl, whereas, the sample volume can range up to 25 µl in the case of dPCR. This ability to process larger volumes allows improved detection of rare targets in plasma samples of patients. The increased sensitivity of 98% observed in our study, compared to the previous ddPCR studies, which showed detection rates ranging from 62%-87%^8,10,11,18^, suggests a significant advantage of employing a 20 µl sample volume per run in dPCR. The copies per ml in our study (mean 1853.9 ± 2948.6 and median = 850 copies/ml plasma (range 0.0-13584),) was also higher compared to previous studies which deployed a ddPCR platform like Cabel et al. (median HPV-ctDNA level 33 copies/ml)^8^, Jeannot et al. in 2016 (mean 1360 ± 2003 copies/ml)^12^ and Jeannot et al. in 2021 (median 42 copies/mL)^6^.

Another major disadvantage reported by previous studies is the inability of droplet digital PCR in detecting early stages. Bønløkke et al., in a cohort of 30 early-stage cervical cancer, were able to detect HPV ctDNA only in 3 cases by ddPCR, demonstrating a sensitivity of 10% for the detection for early-stage cervical cancer^20^. Though we had only 4 patients in stage I, we could detect HPV ctDNA in all 4 patient cfDNA samples (range 42–404.4 HPV copies per ml of plasma). However, a larger number of early-stage cervical cancer cases needs to be analysed by this dPCR assay to confirm its sensitivity in detecting cervical cancers at early stages.

Another major finding of our study was the ability to detect HPV ctDNA in patients with relapse. In most of the patients who did not experience relapse, we observed a significant reduction in HPV ctDNA levels throughout treatment and follow-up, ultimately reaching undetectable levels by 3 months after brachytherapy. This trend suggests that a decline in HPV ctDNA levels may be indicative of HPV clearance due to an effective treatment response, further supporting the utility of HPV ctDNA as a dynamic biomarker for monitoring therapeutic outcomes in HPV-associated cancers, similar to our previous findings in HPV associated Head and Neck cancer^21^. We detected positive HPV ctDNA in 4 patients with relapse, highlighting the potential of monitoring HPV ctDNA levels in predicting relapse. HPV ctDNA detection in post treatment and follow-up samples in these patients suggests that ctDNA based treatment monitoring may provide a real-time, non-invasive approach to detecting minimal residual disease before emergence of clinical or radiological relapse. Our findings align with the previous studies demonstrating the prognostic value of HPV ctDNA in disease surveillance. Our data also suggests that high levels of HPV ctDNA in plasma during therapy might be associated with an increased risk of relapse^18^. This rise in HPV copy numbers at 3 months in one of our patients could have served as an early warning signal, preceding the formal clinical confirmation of disease relapse by traditional testing modalities. Regular ctDNA monitoring could potentially be used as an early warning for the identification of treatment failure before conventional clinical and imaging assessments. A similar study by Jeannot et al. (2021) also reported that patients with persistent HPV ctDNA in serum relapsed within a median time of 10 months (range 2–15 months) from the time of HPV ctDNA detection^6^, thus strengthening the evidence for using HPV ctDNA for monitoring relapse. Overall, while our findings support the potential clinical relevance of HPV ctDNA quantification, confirmation in larger patient cohorts, particularly with respect to relapse or recurrence, will be important to further establish its clinical utility.

The main limitations of the study were varied sampling frequencies and timepoints (different numbers and frequencies of blood draws among patients) during therapy and follow-up, which could not portray a complete picture of cfDNA dynamics during treatment and monitoring of cervical cancer patients. It was also difficult for us to establish a consistent trend for drawing definitive conclusions on monitoring at definitive timepoints. A study by Han et al. compared the accuracy of FDG PET with HPV ctDNA for predicting relapse in patients and observed that the HPV ctDNA test was more accurate than FDG PET in detection of relapse^7^. Larger, prospective studies with well-defined follow-up intervals are needed to validate the clinical utility of HPV ctDNA as a biomarker for treatment response, minimal residual disease detection, and early relapse prediction, and additionally, explore the biological significance of residual HPV cfDNA and its role in long-term outcomes. Incorporating standardized and longitudinal ctDNA testing schedules aligned with routine imaging and clinical follow-up could be useful in determining the utility of HPV ctDNA kinetics in monitoring treatment response and recurrence. This would also allow evaluation of the timing of molecular detection relative to radiological recurrence, which is critical for surveillance strategies. Additionally, future studies with larger cohorts with multiple longitudinal follow-up samples are required to further explore the utility of HPV ctDNA in cervical cancer and provide a well-defined timeline for sample collection for monitoring, defining optimal time points crucial for disease surveillance, leading to better treatment outcomes.

## Methods

### Patient recruitment and sample collection

Eighty seven cervical cancer patients visiting Manipal Comprehensive Cancer Care Centre, Kasturba Medical College, Manipal, were screened for the study between 2020 and 2023. The study was conducted in accordance with the principles of the Declaration of Helsinki. Written informed consent was obtained from all the participants. Ethical approval was obtained from the Institutional Ethics Committee, Kasturba Medical College, Manipal, Manipal Academy of Higher Education, Manipal, India (IECNo. 774/2019), and the study protocol was registered at The Clinical Trials Registry- India (CTRI - CTRI/2020/01/022862) (registered on 20 January 2020). The inclusion criteria were newly diagnosed cases of cervical cancer between age 18 and 80 from the International Federation of Gynecology and Obstetrics (FIGO) stage I–IV. Clinical parameters like age, histological grade, etc., were obtained from the medical records. Twenty healthy adult volunteers without a history of any malignancy were recruited as negative controls.

Tumor biopsy sample was collected at baseline before treatment from each patient. These samples were snap-frozen using ethanol-dry ice bath or liquid nitrogen and stored at −80 °C for further analysis. 10 ml of peripheral whole blood was collected from each patient using BD Vacutainer K2 EDTA tubes at baseline, end of external beam radiation therapy (EBRT), end of brachytherapy and during follow up visits at 1,3,6, 12 and 24 months. 10 ml of whole blood was collected using BD Vacutainer K2 EDTA tubes from the healthy volunteers at the time of recruitment.

The blood samples were processed within 1 h of sample collection. Plasma was separated from blood using a double centrifugation protocol. EDTA blood samples were first centrifuged at 800 × *g* for 10 min at room temperature. Plasma was transferred into 2 ml DNA LoBind Tubes (Eppendorf). To ensure the removal of any residual blood cells, an additional centrifugation at 16,500 × g for 10 min at 4 °C was performed. The supernatant was separated and stored at −80°C until cfDNA extraction.

Samples were thawed on ice and 4 to 5 ml of plasma was used for cfDNA extraction using the QIAamp® Circulating Nucleic Acid Kit (QIAGEN, Hilden, Germany). Purified cfDNA was eluted in 50 µl of elution buffer provided by the kit and stored at −80 °C until further analysis. The concentration of cfDNA was measured using Qubit 1X dsDNA HS Assay Kit on Qubit 4 Fluorometer (Thermo Fisher Scientific, Waltham, MA, USA). cfDNA quality was accessed using a Bioanalyzer 2100 (Agilent) with Agilent High Sensitivity DNA Kit.

### Primer and probe design

HPV 16 AND 18 *E6* gene specific primers and probes were adapted from Damerla et al.^21^. HPV 31 primers and Human GAPDH primers and probes were adapted from previously published studies by Damin et al.^22^, and Svobodová et al.^23^. Probes for HPV 31 were designed using Primer 3 software. The specificity of all the primers and probes was checked using the online NCBI BLASTn tool against the human genome (https://blast.ncbi.nlm.nih.gov/).

Hydrolysis probes for HPV 16, 18, and 31- *E6* gene and *GAPDH* were labelled on the 5′-end with FAM, Cy3, CY5, and HEX fluorescent dyes, respectively. The 3′-end of the probes was labelled with BHQ 1 (Black Hole Quencher) for HPV 16 and *GAPDH* and BHQ 2 for HPV 18 and 31 probes. Primer and probe sequences along with their lengths are presented in Table 2. Primers were manufactured by BioServe, India, and probes were manufactured by Barcode Biosciences, India.

**Table 2:** List of Primers and probes used for dPCR assay

Name	Sequence (5’-3’)	Length (bp)
HPV 16 F	TATGCACAGAGCTGCAAACA	20
HPV 16 R	GCAAAGTCATATACCTCACGTC	22
HPV 16 Probe	**FAM**-TGTGTGTACTGCAAGCAACAGTTACTG-**BHQ1**	27
HPV 18 F	ATGGCGCGCTTTGA	14
HPV 18 R	CTGTAAGTTCCAATACTGTCTTG	23
HPV 18 Probe	**CY3**-CGACCCTACAAGCTACCTGA-**BHQ2**	20
HPV 31 F	GAAATTGCATGAACTAAGCTCG	22
HPV 31 R	CACATATACCTTTGTTTGTCAA	22
HPV 31 Probe	**CY5**-ACCCTACGATGAACTAAGATTGA-**BHQ2**	23
GAPDH F	CCCCACACACATGCACTTACC	21
GAPDH R	CCTAGTCCCAGGGCTTTGATT	21
GAPDH Probe	**HEX**-AAAGAGCTAGGAAGGACAGGCAACTTGGC-**BHQ-1**	29

*FAM* 5(6)-carboxyfluorescein, *CY3* trimethinecyanine, *Cy5* pentamethinecyanine, *HEX* Hexachlorofluorescein, *BHQ* Black Hole Quencher.

### HPV typing

DNA from snap frozen tumor samples was extracted using DNeasy Blood and Tissue Kit (QIAGEN, Hilden, Germany) following the manufacturer’s protocol. Tumor HPV status was determined by using HPV L1 gene amplification, performed by using a two-step PCR approach consisting of a primary amplification with the consensus MY09/MY11 primers followed by a nested PCR with the GP5+/GP6+ primer set. For the primary PCR, a 20 μL reaction mixture was prepared containing 10 μL of EmeraldAmp® GT PCR Master Mix (2× Premix), 1 μL each of MY09 and MY11 primers (10 μM), 2 μL of DNA template (10–50 ng/μL), and nuclease-free water to volume. Thermocycling was carried out for 40 cycles of 95 °C for 30 s, 51 °C for 30 s, and 72 °C for 30 s. Two microliters of the primary PCR product were then used as template for nested PCR with the GP5+/GP6+ primers. The reaction mixtures contained 10 μl of EmeraldAmp® GT PCR Master Mix (2X Premix) (Takara Bio), 1 μl each of forward and reverse primer at 10 μM concentration, DNA templates (2 μl of 10–50 ng/μl concentration), and molecular grade nuclease-free water to make it up to 20 μl. The thermal cycling step included 40 cycles of 95 °C for 30 s, 51 °C for 30 s, and 72 °C for 30 s. The PCR products were run on a 2% agarose gel and imaged on an iBright FL1500 Imaging System (Thermo Fisher Scientific, Waltham, MA, USA). PCR products were purified by FavorPrep GEL/PCR Purification Kit (Favorgen Biotech).

Sanger sequencing of L1 gene PCR products (with GP5+/GP6+ primers) was performed for all the samples, and sequences were compared with known HPV types using the Basic Local Alignment Search Tool for HPV typing. MEGA 11 software was used to align the sequences were aligned to the reference. The sample tested negative for both MY09/MY11, and GP5+/GP6+ PCR was considered negative.

HPV 16, 18, or 31 E6 gene-specific PCR was performed for confirmation of HPV genotype of the samples. Each PCR mixture consisted of 1 μl of each 10 μM forward and reverse primer, 50 ng DNA, 10 μl of EmeraldAmp® GT PCR Master Mix (2X Premix) (Takara Bio), and nuclease-free water to make a final volume of 20 μl. Amplifications were done for 35 cycles, the same as GP5+/GP6+ PCR. The PCR products were run on a 2% agarose gel for visualisation.

### HPV recombinant plasmids

Recombinant HPV plasmids were generated by cloning HPV 16, 18, and 31 E6 sequences into plasmids. DNA extracted from HPV positive cell lines HeLa and SiHa, and a patient tumor sample positive for HPV 31, were used for performing PCR, and subsequently the fresh PCR products were cloned into the pMD20 T-vector (Takara Bio) or pCR4-TOPO vector (Thermo Fisher Scientific), according to the instructions provided by the manufacturers. These recombinant plasmids were 10-fold serially diluted for generating standard curve on qPCR and dPCR.

### qPCR Primer standardisation

To optimise E6 primers, PCR was performed on the QuantStudio 5 Real-Time PCR System. PCR master mixture comprised 12.5 μL of 2X TB Green Premix Ex Taq II (Tli RNaseH Plus) Master Mix (Takara Bio, Inc.), 1 μL of plasmid template, 9.5 μl of nuclease-free water, and 1 μL (10 μM) of each primer (HPV 16/HPV 18/HPV 31). Amplification conditions included an initial denaturation step at 95 °C for 30 s, followed by 40 cycles at 95 °C for 15 s, 51 °C for 30 s, and 72 °C for 30 s. Melting curve analysis was performed using QuantStudio Design and Analysis software (ThermoFisher Scientific). Standard curves were generated by plotting the threshold cycle values against the logarithm of the copy numbers of plasmid DNA standards (serial 10-fold dilution of 10^10^, 10^8^, 10^6^, 10^4^, and 100 copies of HPV16 E6, HPV18 E6, or HPV 31 E6 plasmids) (supplementary Fig. 10).

### dPCR Primer standardisation

The dPCR assay was optimized using QIAcuity EvaGreen (EG) PCR Kit on QIAcuity™ Digital PCR System (QIAGEN; Hilden, Germany). Each dPCR reaction contained 14 μl of 3X EvaGreen PCR Master Mix (QIAGEN, Hilden, Germany), 2 μL (10 μM) of each primer (HPV 16/HPV18/HPV 31), 1 μL template (10^4^ to 10^0^ copies of HPV plasmid), and 21 μL nuclease-free water. The cycling conditions were 95 °C for 2 min, 40 cycles of 95 °C denaturation for 15 s, 51°C annealing for 30 s, and elongation at 72 °C for 30 s. The data was analysed using QIAcuity® Software Suite Version 2.5.0.1. The experimental and calculated copy numbers were plotted in a graph (Supplementary Fig. 11).

### Uniplex qPCR development

Quantitative real-time PCR (qPCR) was performed to optimise the annealing temperatures of the primer-probe sets for dPCR. A range of annealing temperatures, 51–63 °C, was tested on 10^4^ copies/μl of HPV recombinant plasmids. The reaction mixture comprised Premix Ex Taq™ (Probe qPCR), ROX Plus (RR390A) (Takara Bio). 25 μl reactions were set up according to the instructions of the Premix Ex TaqTM (Probe qPCR) kit as follows: 0.5 μl each of the forward and reverse primer, 1 μl of probe, 1 μl of template (10^4^ copies/ μl of HPV plasmids), 12.5 μl of Premix Ex Taq^TM^ (Probe qPCR), and 9.5 μl of water. PCR assays were performed using the QuantStudio 5 Real-Time PCR System. The amplification was performed at 95 °C for 30 s and 40 cycles of 95 °C for 5 s, (51–63) °C for 10 s, and 72 °C for 1 min.

### Multiplex qPCR optimisation

To evaluate the specificity of the multiplex system, the primer-probe mixture of each target was tested containing serial dilutions of HPV E6 plasmids carrying 10,000, 1000, 100, 10, and 1 copies plasmids in a background of extracted genomic DNA. The following combinations were used to assess potential cross-reactivity.

For HPV 16 detection: HPV 16 plasmids in the background of HeLa cell-extracted DNA and HPV 31 genomic DNA.

For HPV 18 detection: HPV 18 plasmids in the background of SiHa cell-extracted DNA and HPV 31 genomic DNA.

For HPV 31 detection: HPV 31 plasmids in the background of SiHa cell-extracted DNA and HeLa cell-extracted DNA.

Each reaction was performed under the same amplification conditions as described for the multiplex assay. All experiments were performed in triplicate. GAPDH was not used in these PCR master mixes as housekeeping control.

### Uniplex digital PCR optimisation

dPCR was performed to optimise the concentration primers and probes using QIAcuity™ Digital PCR System (QIAGEN; Hilden, Germany) according to the manufacturer’s instructions. The recombinant pmD20T and Topo PCR4 plasmids containing nucleotide sequences of HPV 16 E6, HPV 18 E6, and HPV 31 E6 were used as positive controls. for assay validation. For the optimisation experiment, the dPCR reaction contained 10 μl of 4x Probe PCR Master Mix (QIAcuity Probe PCR Kit (QIAGEN, Hilden, Germany)), 2 μl each of forward and reverse primer, and 4 μl probe, 1 μl of template (200 copies of HPV plasmid), and nuclease-free water to 40 μL. Primer and probe concentrations were tested at three final concentration combinations, corresponding to primer:probe ratios of 0.5:0.5, 1.0:1.0, and 1.5:1.5 μM. The 26k- 24 well nanoplate was sealed and loaded on the QIAcuity One dPCR. The cycling conditions were PCR initial heat activation at 95 °C for 2 min, 40 cycles of 95 °C denaturation for 15 s and 55 °C annealing and elongation for 1 min. Assay performance for each primer:probe condition was evaluated based on cluster separation, fluorescence amplitude, amount of “rain”, and concordance between measured and expected copy number. The primer:probe concentration giving the best combination of clean positive/negative cluster separation, minimal rain, and accurate quantification was selected.

### Multiplex dPCR optimisation

The optimized primer–probe concentrations were then applied to multiplex dPCR. Equimolar pools of forward primers, reverse primers, and probes (each at a final concentration of 1.5 µM for HPV16, HPV18, HPV31, and GAPDH) were prepared and used directly in the reaction mixture.

### dPCR cross reactivity determination

To assess cross-reactivity in the multiplex HPV assay, we made defined matrix mixes using genomic DNA from established cell lines and a validated HPV31-positive DNA sample as templates. SiHa (HPV16-positive) and HeLa (HPV18-positive) cell line DNA, and the HPV31-positive DNA sample, were quantified and diluted. For cross-reactivity testing, we prepared the following sets of templates:three omission mixes in which one genotype was intentionally excluded (SiHa + HeLa, lacking HPV31; SiHa + HPV31, lacking HeLa; HeLa + HPV31, lacking SiHa). No-template controls (NTCs) were included to monitor contamination.

The reaction contained 10 μL of 4× Probe PCR Master Mix, 2 μL each of forward-primer and reverse-primer pools, 4 μL of probe pool, 1 μL of EcoRI-HF® (NEB®) restriction enzyme, 2 μL of template mix, and nuclease-free water to make reaction up to 40 μL. Samples were loaded onto a 26k-24 well nanoplate and sealed. The plate was incubated at room temperature for 10 min following manufacturer’s protocol. Enzymatic fragmentation of genomic DNA was performed to ensure even distribution of templates throughout the QIAcuity Nanoplate as suggested by manufacturer. The plate was then loaded on the QIAcuity One dPCR post-incubation. The cycling conditions optimised in the previous step were used.

### Limit of blank and Limit of detection

The LoB and LoD were established following the EP17 guideline, Protocols for Determination of Limits of Detection and Limits of Quantitation, issued by the Clinical and Laboratory Standards Institute^24^. The LoB was determined by testing a series of 20 blanks (reactions in which the target template is excluded, and nuclease-free water is added instead). The results from these 20 blanks were used to calculate LoB using the equation:

5 $${\rm{LoB}}={\mathrm{mean}}_{\mathrm{blank}}+1.645\times {\mathrm{SD}}_{\mathrm{blank}}$$

where ${\mathrm{mean}}_{\mathrm{blank}}$ represents the mean number of positive partitions observed in blank (no-template control) reactions, and ${\mathrm{SD}}_{\mathrm{blank}}$ denotes the corresponding standard deviation.

To determine the LoD, serial dilutions of the HPV plasmid were prepared and tested across the low-copy range (10^3^ to 10^0^ copies per reaction). LoD was calculated using the following equation:

6 $$\mathrm{LoD}=\mathrm{LoB}+1.645\times {\mathrm{SD}}_{{\mathrm{low}}\,{\mathrm{concentration}}\,{\mathrm{sample}}}$$

where LoB denotes the limit of blank, and ${\mathrm{SD}}_{{\mathrm{low}}\,{\mathrm{concentration}}\,{\mathrm{sample}}}$ represents the standard deviation of measurements obtained from low-concentration HPV DNA samples.

### Diagnostic performance of HPV16/18/31 multiplex dPCR on clinical cfDNA samples

The standardized dPCR protocol was applied to quantify HPV cfDNA in clinical plasma cfDNA samples, using GAPDH as a housekeeping/internal control. Patients who had a tumor biopsy and blood sample available at diagnosis and prior to treatment were included in the diagnostic analysis. Participants who had longitudinal follow-up samples constituted the prospective cohort used for treatment monitoring. Individuals with metastatic disease were excluded from this cohort, as metastatic cases have a different treatment regimen as compared to non-metastatic cervical cancer cases.

The dPCR reaction composition remained the same, except for the use of 20 μL of cfDNA sample instead of 1 μL of plasmid DNA. Specifically, the reaction contained 10 μL of 4× Probe PCR Master Mix, 2 μL each of forward-primer and reverse-primer pools, 4 μL of probe pool, 20 μL of cfDNA sample, and 2 μL of nuclease-free water. Each plate also included a no-template control, where nuclease-free water was used instead of the template. The 26k- 24-well nanoplate was sealed and loaded onto the QIAcuity One dPCR system. The cycling conditions remained unchanged: initial heat activation at 95 °C for 2 min, followed by 40 cycles of 95 °C denaturation for 15 s and 55°C annealing/elongation for 1 min. QIAcuity® Software Suite Version 2.5.0.1 was used for visualising partitioning, processing, and fluorescent signal processing. The number of copies per ml of plasma was calculated as per the formula given by Qvick et al.^25^.

7 $$\frac{{\mathrm{Copies}}}{{\mathrm{mL}}}plasma=\frac{\frac{Copies}{\mu L}X\,Reaction\,volume\,X\,elution\,volume}{Sample\,volume\,in\,dPCR\,reaction\,X\,Plasma\,volume}$$

### Statistical analysis

Initial analysis of the QIAcuity data was conducted using the QIAcuity Software Suite v.2.5.0.1. Sensitivity was defined as the number of pretreatment samples tested positive for HPV16, 18, or 31-cfDNA from plasma divided by the number of samples with HPV16, 18, 31-detected on tumors. Specificity was defined as the number of samples tested negative for HPV16, 18, or 31-cfDNA from plasma divided by the number of samples with HPV16, 18, 31-detected on tumors. Kruskal–Wallis test or unpaired *t*-test was used for comparison of variables. The GraphPad Prism v-10 software (GraphPad Software, San Diego, CA, USA) was used for further analyses.

## Supplementary information

Supplementary Table 1

Supplementary Figures.

## Data Availability

The data that support the findings of this study are available from the corresponding author upon reasonable request.
