# Supplementary material for: Nanoplate based digital PCR assay for effective quantification of plasma HPV circulating tumor DNA
Source: NPJ Precis Oncol. 2026 Apr 21;10:242. doi: 10.1038/s41698-026-01424-y (PMC13284192; doi:10.1038/s41698-026-01424-y)
Supplement: Supplementary file 1 — Supplementary Table 1 [file 41698_2026_1424_MOESM1_ESM.pdf]

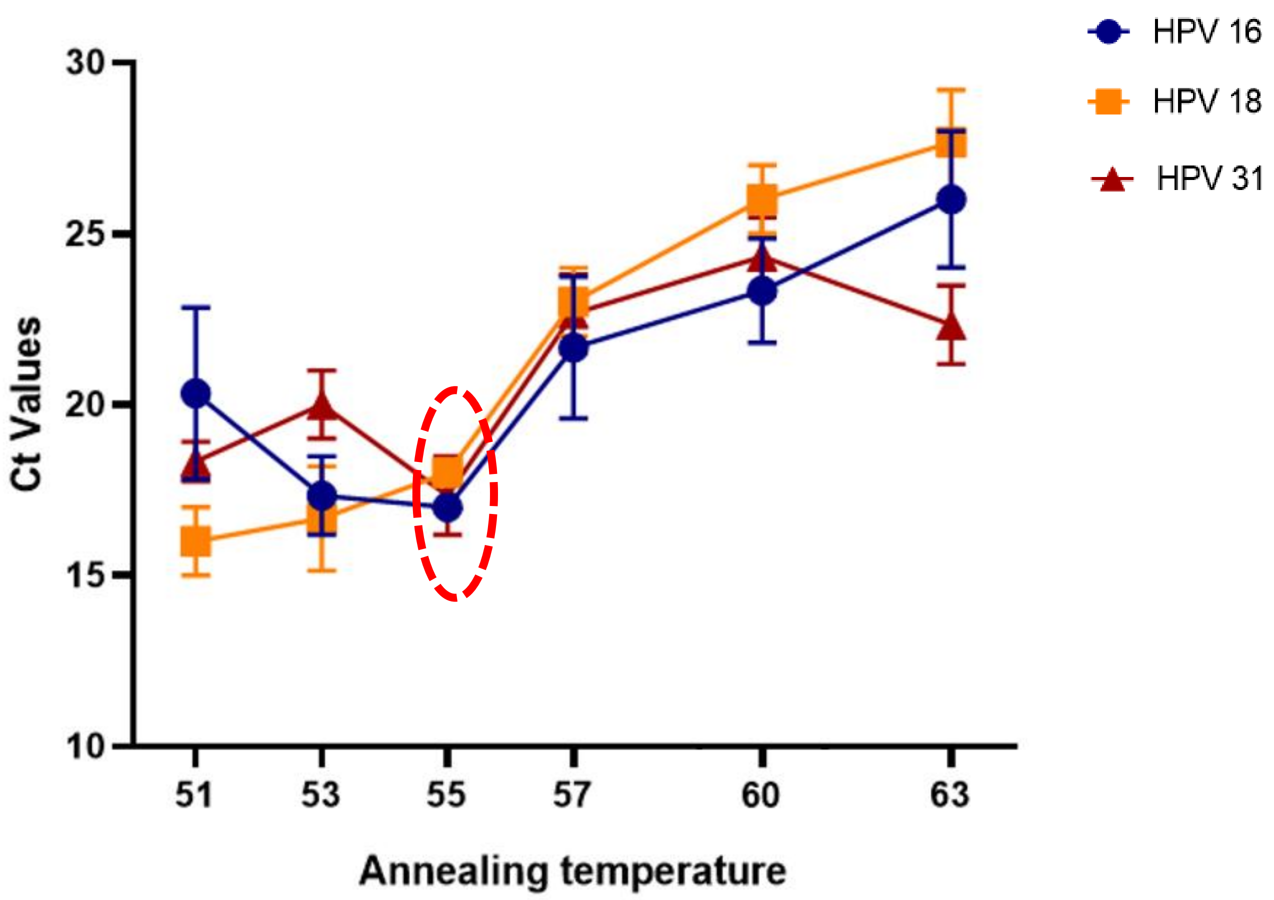

Supplementary Figure 1 : Optimization of conditions for qPCR. Optimization of annealing temperature for HPV primers and probes using 10^4 copies/µL HPV16 (blue circle), 18 (orange square) and 31 (red triangle) plasmids. Following annealing temperatures: 51, 53, 55, 57, 60, 63°C were used. The optimal annealing temperature was 55°C highlighted by a red circle.

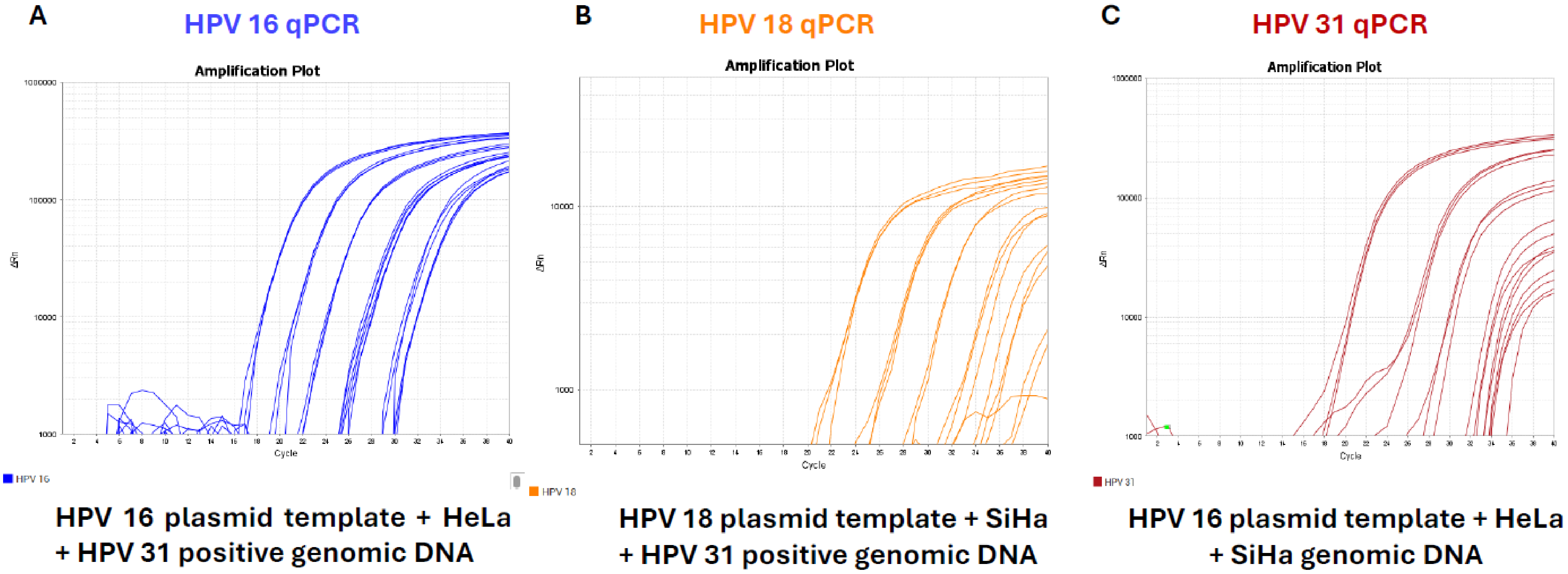

Supplementary Figure 2: Specificity Analysis for HPV Primers and Probes.
qPCR results of serial dilutions of HPV E6 plasmids carrying 10^4, 10^3, 10^2, 10^1 and 10^0 copies plasmids with the background of 30 ng of genomic DNA (HeLa, SiHa and genomic DNA from a patient positive for HPV 31) as sample.
(A) HPV 16 E6 plasmids spiked in 30ng of HeLa cell-extracted DNA and HPV 31 genomic DNA as the background.
(B) HPV 18 E6 plasmids spiked in 30ng of SiHa cell-extracted DNA and HPV 31 genomic DNA were used as the background.
C) HPV 31 E6 plasmids spiked in 30ng of SiHa cell-extracted DNA and HeLa cell-extracted DNA served as the background.

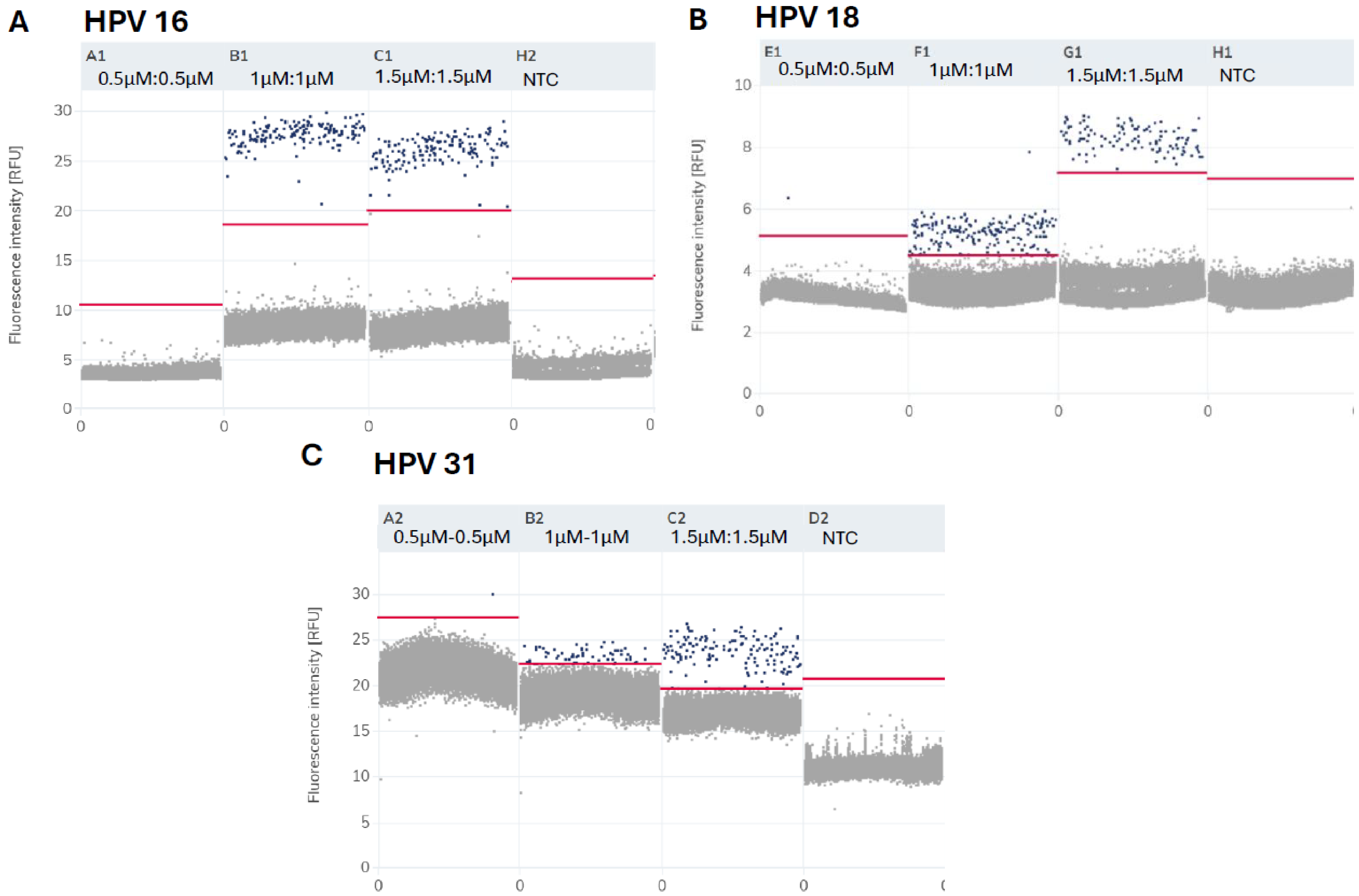

Supplementary Figure 3: Primer–probe ratio optimisation for the multiplex dPCR assay. Primer–probe concentration ratios evaluated to determine the optimal balance between signal strength and cluster separation for each HPV genotype. Panels show fluorescence amplitude plots for (A) HPV16, (B) HPV18, and (C) HPV31 at three concentration combinations (0.5 µM:0.5 µM, 1 µM:1 µM, and 1.5 µM:1.5 µM), alongside no-template controls (NTCs).

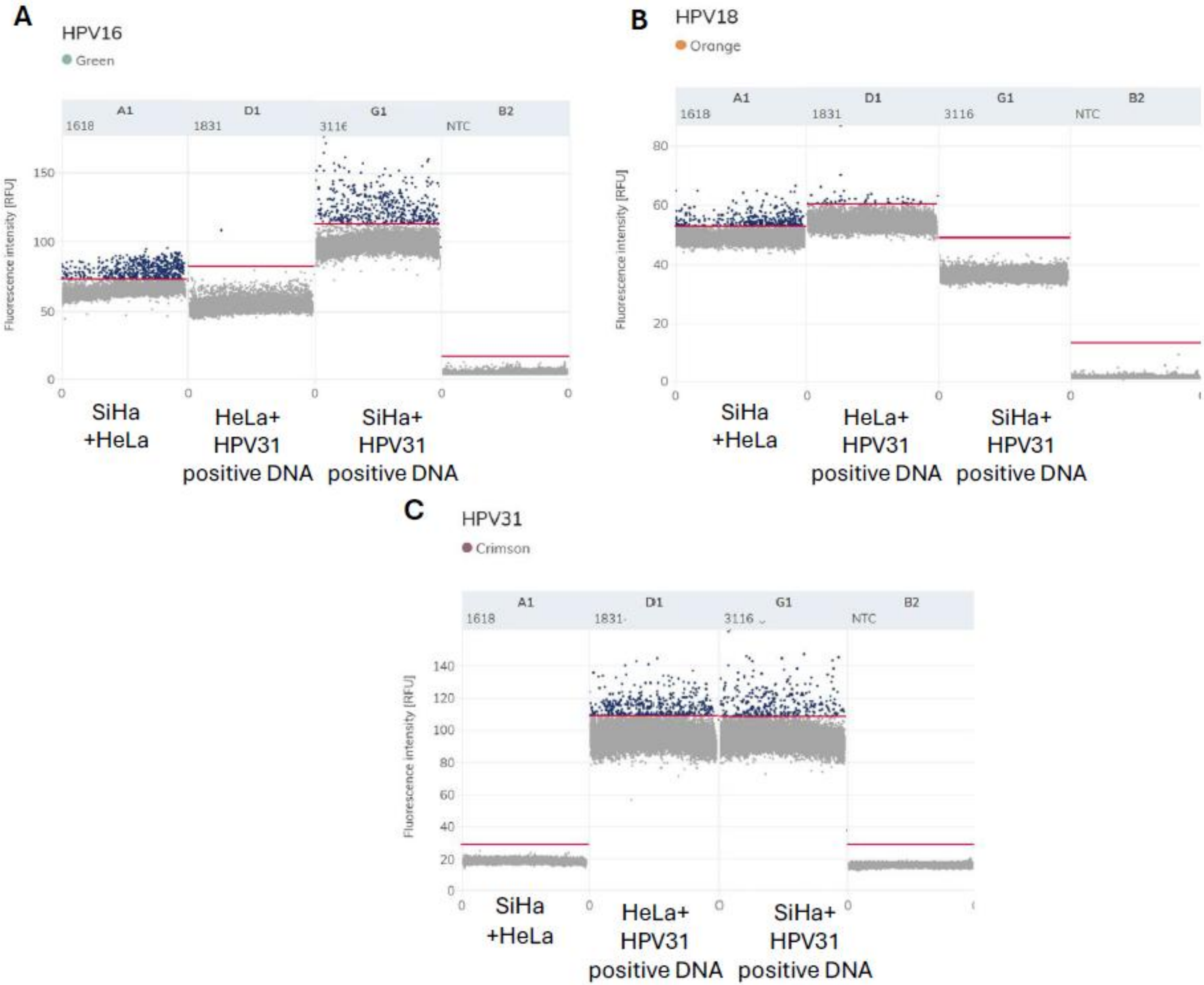

Supplementary Figure 4: Cross-reactivity assessment of the multiplex dPCR assay using omission-matrix mixes. Cross-reactivity evaluated by preparing omission-matrix reactions in which one HPV genotype was intentionally left out while the remaining two genotypes were included. Panels show the fluorescence cluster separation for (A) HPV16, (B) HPV18, and (C) HPV31 across omission-matrix combinations.

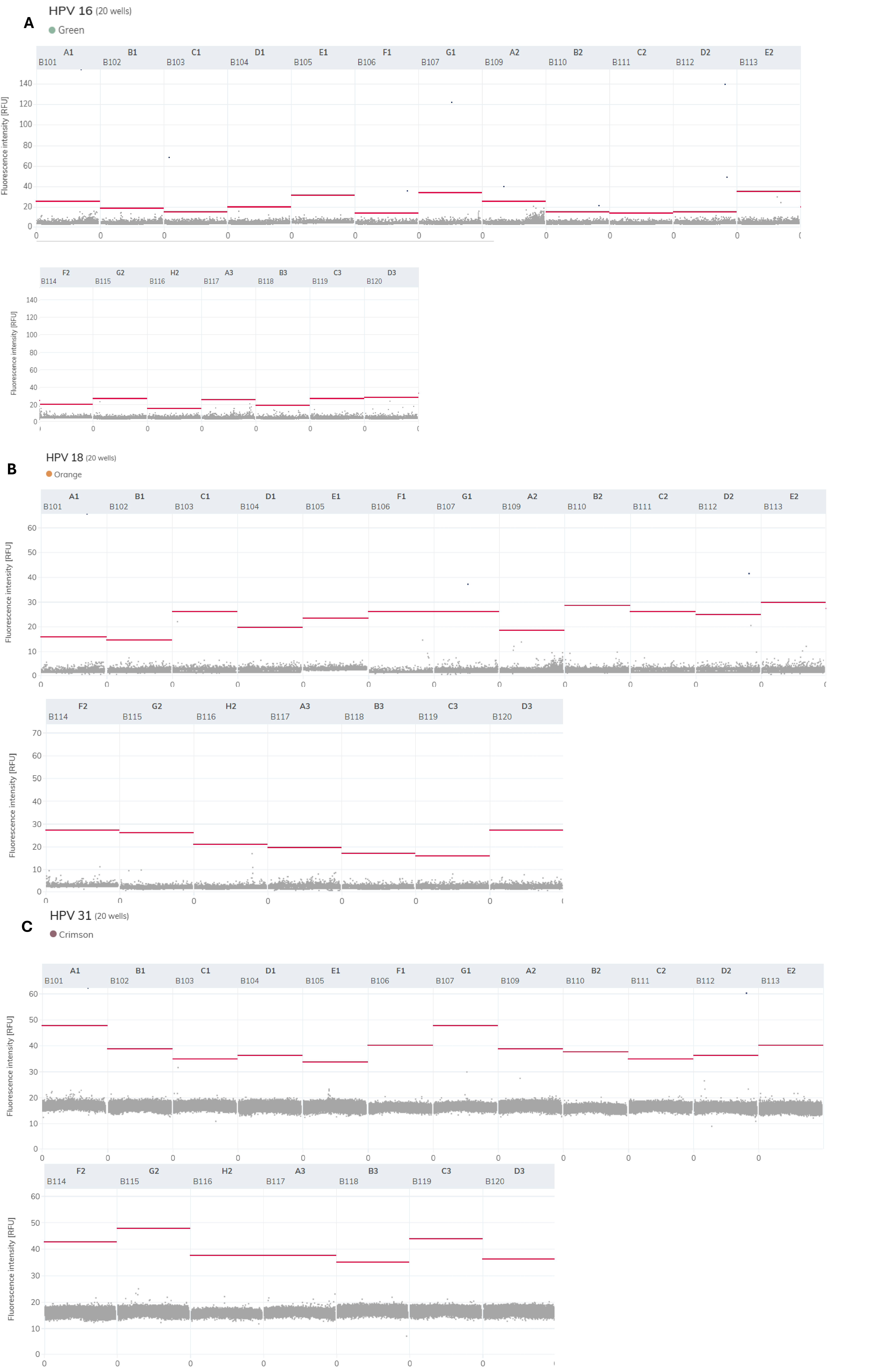

Supplementary Figure 5: Limit of Blank (LoB) assessment for multiplex dPCR assay

LoB was determined for (A) HPV16, (B) HPV18, and (C) HPV31 using 20 DNA-free blank reactions processed under identical assay conditions. Each panel shows the distribution of false-positive droplet counts across replicates.

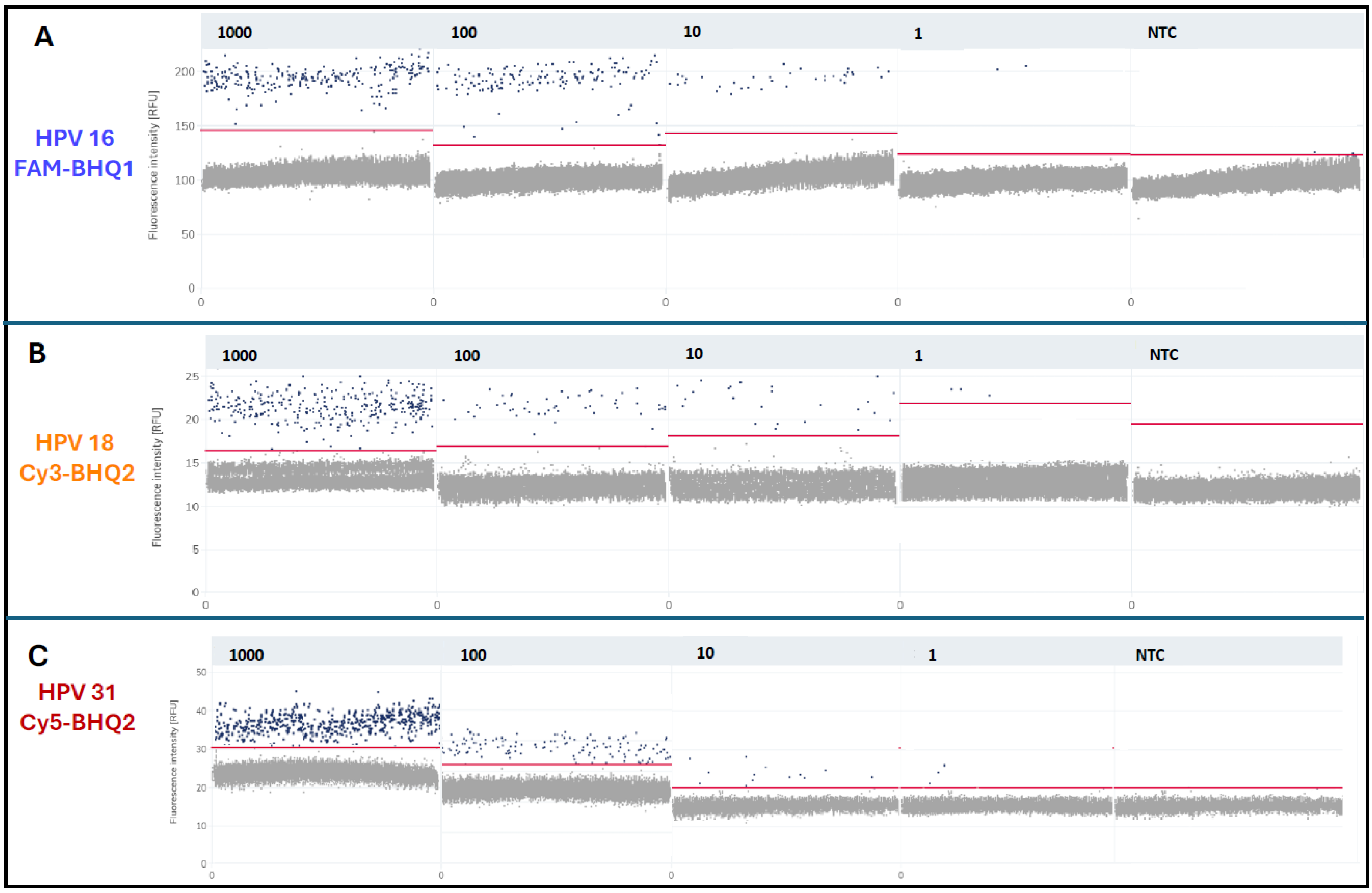

Supplementary Figure 6: Representative quantification of HPV plasmids by dPCR. (Limit of Detection) (A) Copy number of HPV16 plasmid, serially diluted from 10^3 to 10^0 copies/μL and dPCR amplified. (B) Copy number of HPV18 plasmid, serially diluted from 10^3 to 10^0 copies/μL and dPCR amplified. (C) Copy number of HPV31 plasmid, serially diluted from 10^3 to 10^0copies/μL and dPCR amplified.

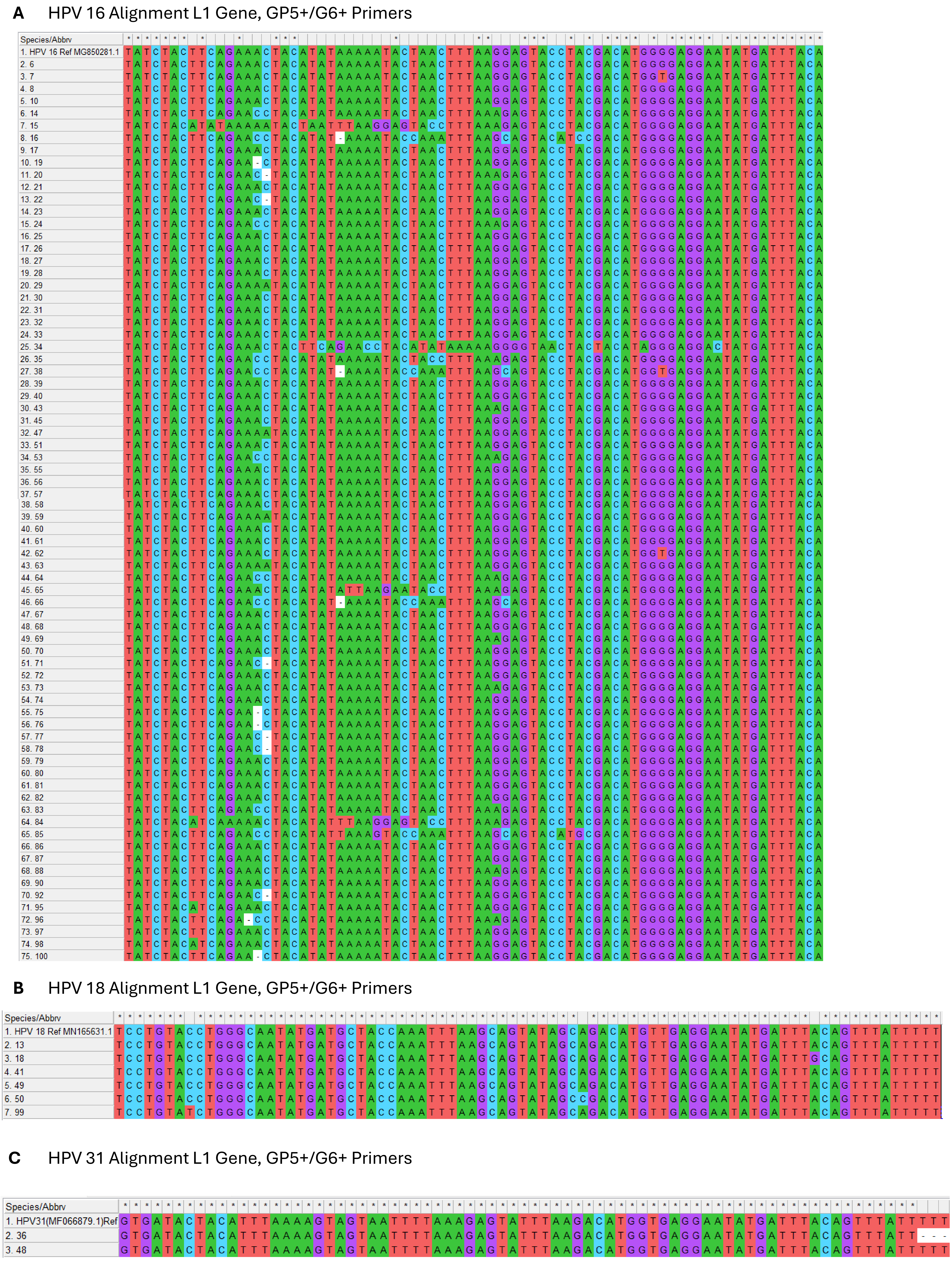

Supplementary Figure 7: Sanger sequencing alignment of HPV L1 gene regions amplified using GP5+/GP6+ primers.

Sequencing analysis was performed by aligning Sanger sequencing reads generated using GP5+/GP6+ primers in MEGA 11. (A) Multiple sequence alignment of HPV16 L1 gene amplicons from patient samples compared with the HPV16 reference sequence. (B) Alignment of HPV18 sequences against the HPV18 reference. (C) Alignment of HPV31 sequences against the HPV31 reference.

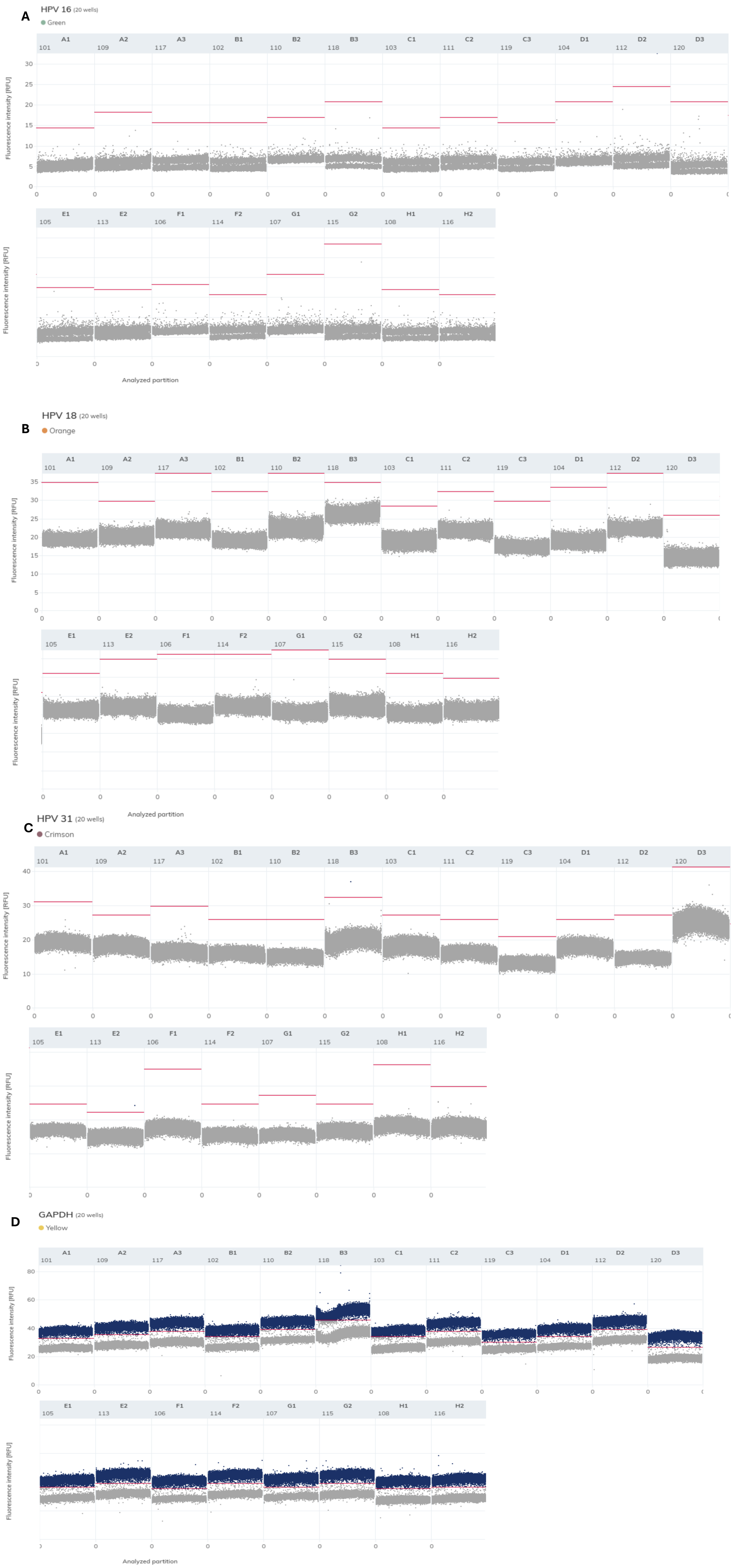

Supplementary Figure 8: dPCR analysis of healthy normal-control samples across all assay targets. Digital PCR performed on plasma from HPV-negative healthy controls to assess the assay specificity. The figure includes four panels showing results for: (A) HPV16, (B) HPV18, (C) HPV31, and (D) GAPDH (human genomic control).

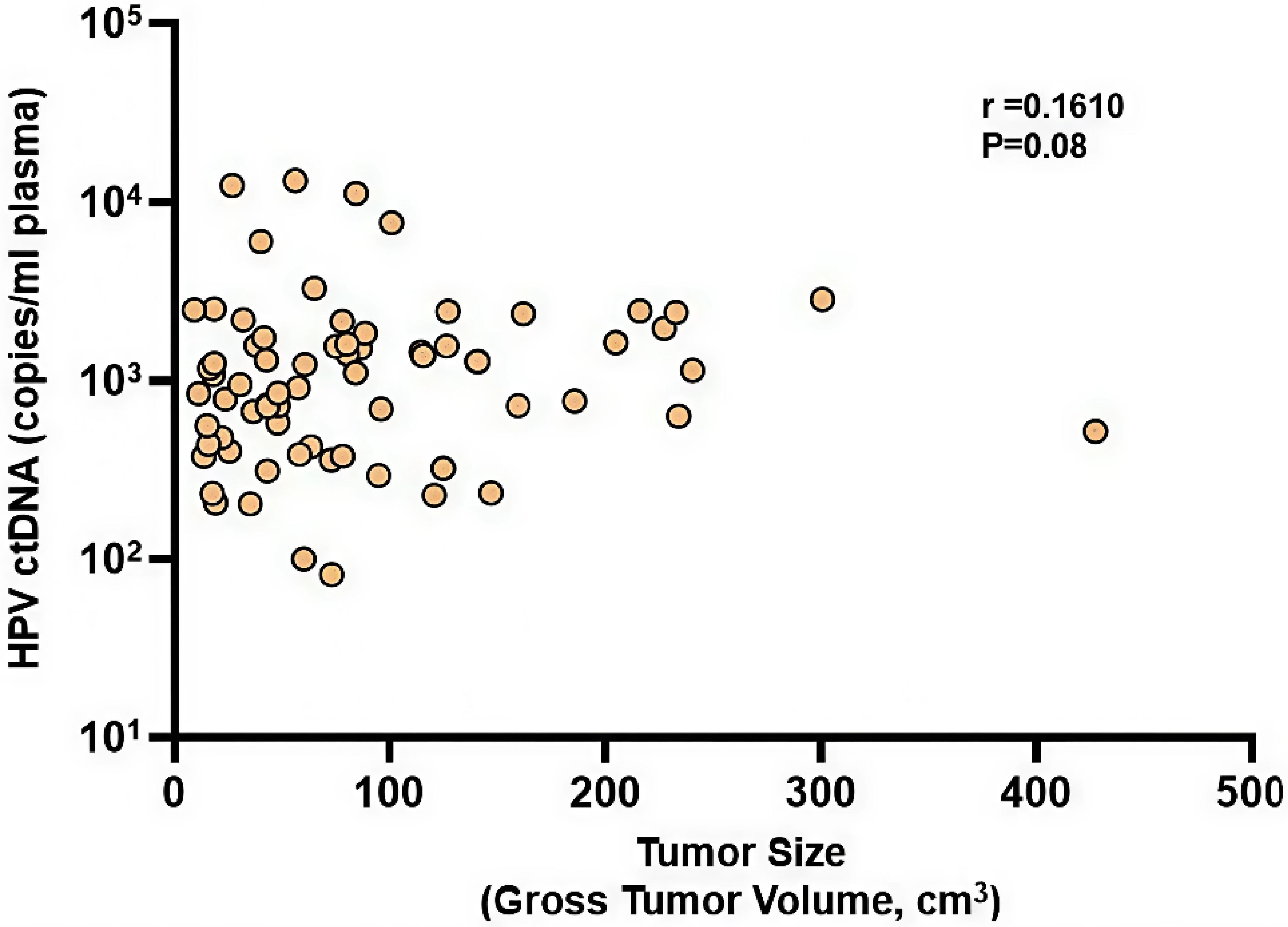

Supplementary Figure 9: Correlation between HPV ctDNA in pretreatment samples and gross tumor volume. Spearman's rank correlation analysis was conducted to examine the relationships between gross tumor volume and HPV copies/ml. The correlation coefficients (rho) observed was 0.1610 (p=0.08)

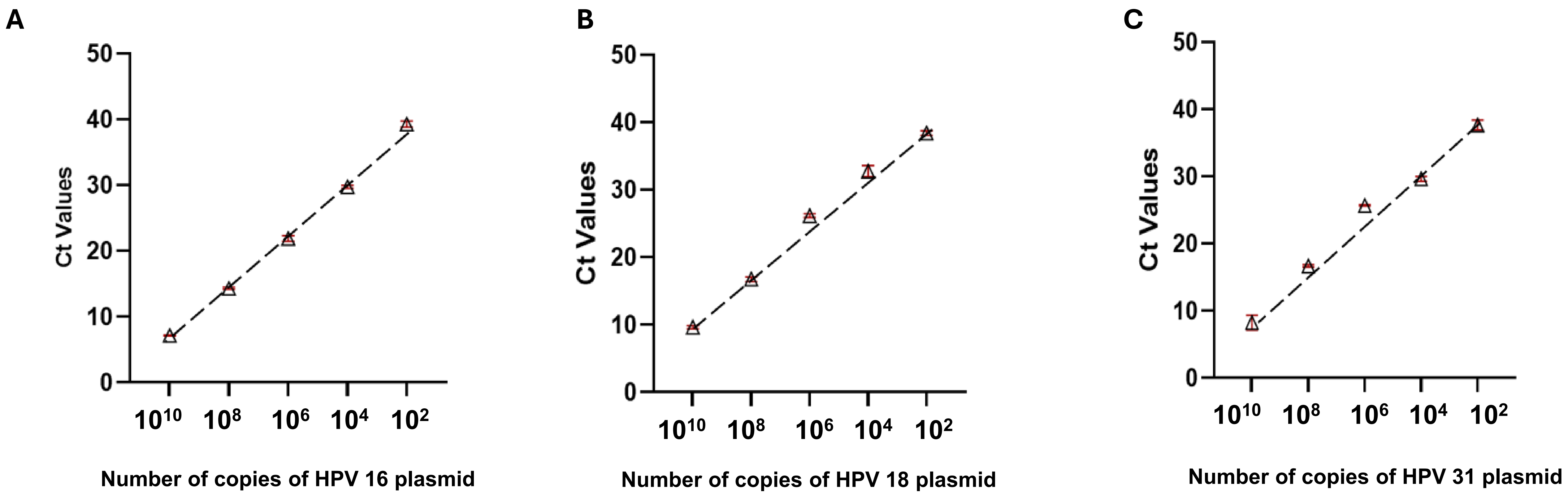

Supplementary Figure 10: Standard curve of E6 plasmids in qPCR using TBgreen ExTaq II (A) Standard Curve for HPV 16 E6 plasmids (serial dilutions 10^{10}, 10^{8}, 10^{6}, 10^{4} and 10^{2} copies of plasmids). (B) Standard Curve for HPV 18 E6 plasmids (serial dilutions 10^{10}, 10^{8}, 10^{6}, 10^{4} and 10^{2}copies of plasmids). (C) Standard Curve for HPV 31 E6 plasmids (serial dilutions 10^{10}, 10^{8}, 10^{6}, 10^{4} and 10^{2} copies of plasmids).

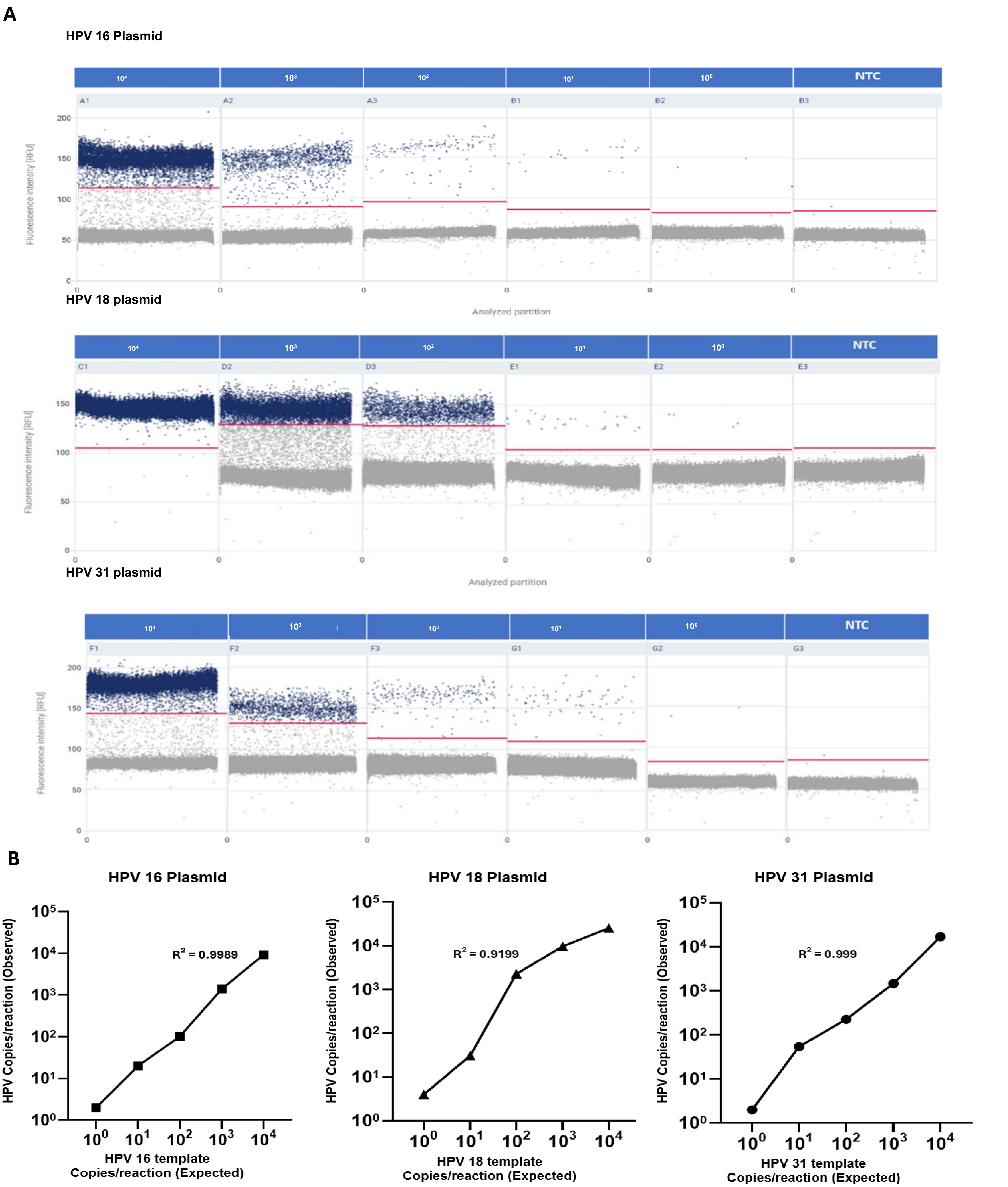

Supplementary Figure 11: Standardisation of HPV E6 plasmids using EvaGreen on dPCR. (A) Representative quantification of HPV (16, 18 and 31) E6 plasmids copy number from 10^4 to 10^0 copy on dPCR using EvaGreen. (B) Graph depicting experimental vs calculated copy numbers HPV (16, 18 and 31) E6 plasmids analysed on dPCR.
