# Supplementary material for: Nanoplate based digital PCR assay for effective quantification of plasma HPV circulating tumor DNA
Source: NPJ Precis Oncol. 2026 Apr 21;10:242. doi: 10.1038/s41698-026-01424-y (PMC13284192; doi:10.1038/s41698-026-01424-y)
Supplement: Supplementary file 2 — Supplementary Figures. [file 41698_2026_1424_MOESM2_ESM.pdf]

Supplementary table 1. Digital PCR assay data using HPV16, 18 and 31 probes in 20 no templat

Well	HPV16 Positive Partitions	HPV18 Positive Partitions	HPV31 itive Partitions
B1	**1**	**1**	**1**
B2	**1**	0	0
B3	0	0	0
B4	0	0	0
B5	**1**	0	0
B6	0	0	0
B7	**1**	0	0
B8	0	0	0
B9	0	0	0
B10	0	0	0
B11	**2**	**1**	**1**
B12	0	0	0
B13	0	0	0
B14	0	0	0
B15	**1**	0	0
B16	0	**1**	0
B17	**1**	0	0
B18	0	0	0
B19	0	0	0
B20	0	0	0
**Mean**	0.4	0.15	0.1
**Stdev**	0.583095189	0.357071421	0.3
**LoB**	1.359191587	0.737382488	0.5935

e controls to determine limit of blank.
